# Latitudinal variation in seasonal cycle mediates population differences in barnacle reproduction phenology

**DOI:** 10.1002/ecy.70415

**Published:** 2026-05-19

**Authors:** Jane B. Weinstock, Jesús Pineda, Claudio DiBacco, Salvatore Genovese, Victoria Starczak, Kira Krumhansl

**Affiliations:** ^1^ MIT‐WHOI Joint Program in Oceanography/Applied Ocean Science & Engineering Cambridge and Woods Hole Massachusetts USA; ^2^ Biology Department Woods Hole Oceanographic Institution Woods Hole Massachusetts USA; ^3^ Fisheries and Oceans Canada Bedford Institute of Oceanography Dartmouth Nova Scotia Canada; ^4^ Division of Natural Sciences and Mathematics, College of General Studies Boston University Boston Massachusetts USA; ^5^ Present address: Alfred‐Wegener‐Institut Helmholtz‐Zentrum für Polar‐ und Meeresforschung Biologische Anstalt Helgoland Helgoland Germany

**Keywords:** brooding, climate change, intertidal, larval dispersal, physiology, temperature, warming

## Abstract

The timing of life history events around reproduction and early development is critical in population dynamics, and it can determine recruitment success, species dispersal, and population connectivity. In ectotherms, as well as in plants and fungi, phenology is mediated by the nonlinear effects of temperature on physiology and development, meaning that spatiotemporal variation in temperature can exert powerful controls on the timing of local reproduction and recruitment. Here, we examine reproduction phenology (fertilization of embryos, duration of embryonic development during brooding, and larval release) of the intertidal acorn barnacle *Semibalanus balanoides* in 2002–2004 and in 2019–2024 at up to eight sites along a steep temperature gradient in the northwest Atlantic Ocean. At each site and year, we assessed how phenology varied with intertidal temperature, estimated with a hybrid atmosphere–ocean data assimilation model. Although within‐site reproduction was delayed due to interannual and decadal fall warming (3.7 days per 1°C), fertilization at all sites in all years still occurred within a 1‐month timeframe. In contrast, latitudinal differences in intertidal temperature resulted in substantially different brooding durations (up to 95 days difference) and, by extension, larval release timing (e.g., Dec 18 vs. Apr 4). Consequently, lower latitude larvae tended to enter the water column at the start of winter, while higher latitude larvae were not released until spring. These different larval release times result in regional differences in temperature‐mediated larval development, potentially resulting in lower latitude populations experiencing greater dispersal. Our study is one of the first to evaluate these relationships through both space and time in natural populations, and we show that both spatial gradients and interannual variation in the seasonal temperature cycle can mediate reproductive physiology and dispersal of temperate and polar species.

## INTRODUCTION

Temperature drives both the timing of reproduction and the rate of early development in plants, fungi, insects, amphibians, and aquatic and marine invertebrates and fishes (Andrew et al., 2018; Fernández‐Pascual et al., 2021; Fischer et al., 2016; Gillooly et al., 2002; Poloczanska et al., 2013). Phenology (i.e., seasonal timing) of species reproduction often exhibits plasticity in response to temperature or temperature‐mediated cues, allowing populations to align with local conditions (e.g., Opdal et al., 2024), likely facilitating population‐wide reproductive synchrony and success (Olive, 1992). Temperature also exerts strong, nonlinear effects on physiology and early development, determining rates of processes ranging from seed germination and fruit maturation in land plants to embryonic and larval development in marine invertebrates, insects, and amphibians (Fernández‐Pascual et al., 2021; Fischer et al., 2016; Gillooly et al., 2002). Species with wide geographic distributions may thus display a spatial mosaic of reproduction and recruitment timing along latitudinal or altitudinal clines, with implications for population and community dynamics.

The timing of reproduction determines the conditions experienced by progeny, which can have outsized impacts on organisms with complex life cycles. Early stages are often life cycle bottlenecks (Gosselin & Qian, 1996; White & Harper, 1970), and in organisms with sessile or slow‐moving adult forms, these stages also encompass the majority of dispersal potential. Realized dispersal, however, is a function of both biophysical transport processes and survivorship (Pineda et al., 2007). Thus, in these organisms, reproduction timing can ultimately impact the degree of propagule or larval exchange between populations and, by extension, genetic structure along the geographic range (Bowen et al., 2016; Palumbi & Pinsky, 2013).

Particularly in high latitudes, many species reproduce seasonally, due to physiological limitations on reproduction, growth, and feeding during winter (Conover, 1992; Pau et al., 2011), and as the climate warms there is evidence of a commensurate shift in reproductive phenology (e.g., Parmesan & Yohe, 2003; Philippart et al., 2003). In temperate terrestrial systems, there has been an advancement of biological spring and delay of fall (Parmesan & Yohe, 2003; Peñuelas et al., 2009). Investigation of the impact of warming on reproduction phenology in marine systems, however, has been more limited and has largely focused on spring‐reproducing seabirds, fish, zooplankton, and phytoplankton (Poloczanska et al., 2013). In aggregate, observed shifts in reproductive phenology follow the expected pattern (earlier reproduction in spring, delayed reproduction in fall), but there is an enormous amount of variation across individual species (Poloczanska et al., 2013; Staudinger et al., 2019). Notably, few studies in any system have extended the concept of seasonal reproduction timing to temperature effects on early development, particularly in naturally varying conditions (though see McGeady et al., 2021). Yet quantifying the rates and impacts of species phenology variability and change across communities is critical, as these may alter species dispersal (Fuchs et al., 2020), with consequences to species interactions and community organization (Zou & Rudolf, 2023).

Here, we examine phenology of the abundant acorn barnacle *Semibalanus balanoides* in the northwest Atlantic, which serves as an ideal system to measure phenology and phenology change over a lengthy reproductive cycle in a rapidly warming temperate region. Individuals are obligate cross‐fertilizing hermaphrodites, producing one brood per year that is held in the mantle cavity over a period of weeks to months (reviewed in Peterson, 1966; Barnes, 1989; Anderson, 1994; Herrera et al., 2021). Fertilization generally takes place in the fall, occurring earlier in more northern populations, and at any given site, timing can vary by as much as 13 days between individuals near the upper and lower limits of the population's vertical range relative to sea level (Crisp, 1959; Davenport et al., 2005; Herrera et al., 2021; Peterson, 1966). Fertilization may be delayed at temperatures <2°C, and it may be inhibited at temperatures >15°C and by exposure to regular periods of illumination ≥12 h (Barnes, 1963). Photoperiod may thus contribute to broad latitudinal trends in fertilization timing (across ~14° of latitude; Davenport et al., 2005). However, because photoperiod at high latitudes is a function of day of year, correlations between raw field observations of fertilization timing and photoperiod may also simply reflect this underlying correlation. Fertilized embryos develop internally until larvae are released in winter or spring (Crisp, 1954; Herrera et al., 2021; Peterson, 1966). Both embryonic and larval development times decrease exponentially with increasing temperature (Barnes & Barnes, 1976; Crisp, 1959; Harms, 1984). Larval release can occur ~1 month after fertilization near the lower latitude end of its range (Herrera et al., 2021), while the northernmost populations might brood embryos for more than 8 months (Peterson, 1966). Depending on temperatures experienced during development, larval duration can range from 10 to 90 days (Barnes & Barnes, 1958; Harms, 1984; Peterson, 1966; personal observation). Such temperature‐driven variability in larval duration suggests that the point at which larval release timing intersects with the seasonal cycle could dramatically impact larval development and dispersal (Scheltema, 1986; Shanks, 2009).

We measured the impacts of intertidal temperature on the start and end of *S. balanoides* reproduction across 760 km (~3° of latitude) and 20 years. This spatial extent encapsulates much of the central range of *S. balanoides* in North America, and it includes three distinct temperature regimes while minimizing differences in fall photoperiod. Because we are most interested in the reproductive processes affecting larval dispersal, we use “reproduction” to refer to population‐level measurements of the female reproductive cycle, with focus on timing of fertilization, length of time spent brooding the developing embryos, and timing of larval release. We ask (1) How do spatially and seasonally varying temperatures impact brooding duration and larval release timing? (2) How does interannual variation in temperature impact the start of reproduction (i.e., fertilization timing), and is there evidence of a temperature‐driven phenology shift due to regional warming over the 20‐year period?

## METHODS & MATERIALS

### Study system

**FIGURE 1:**
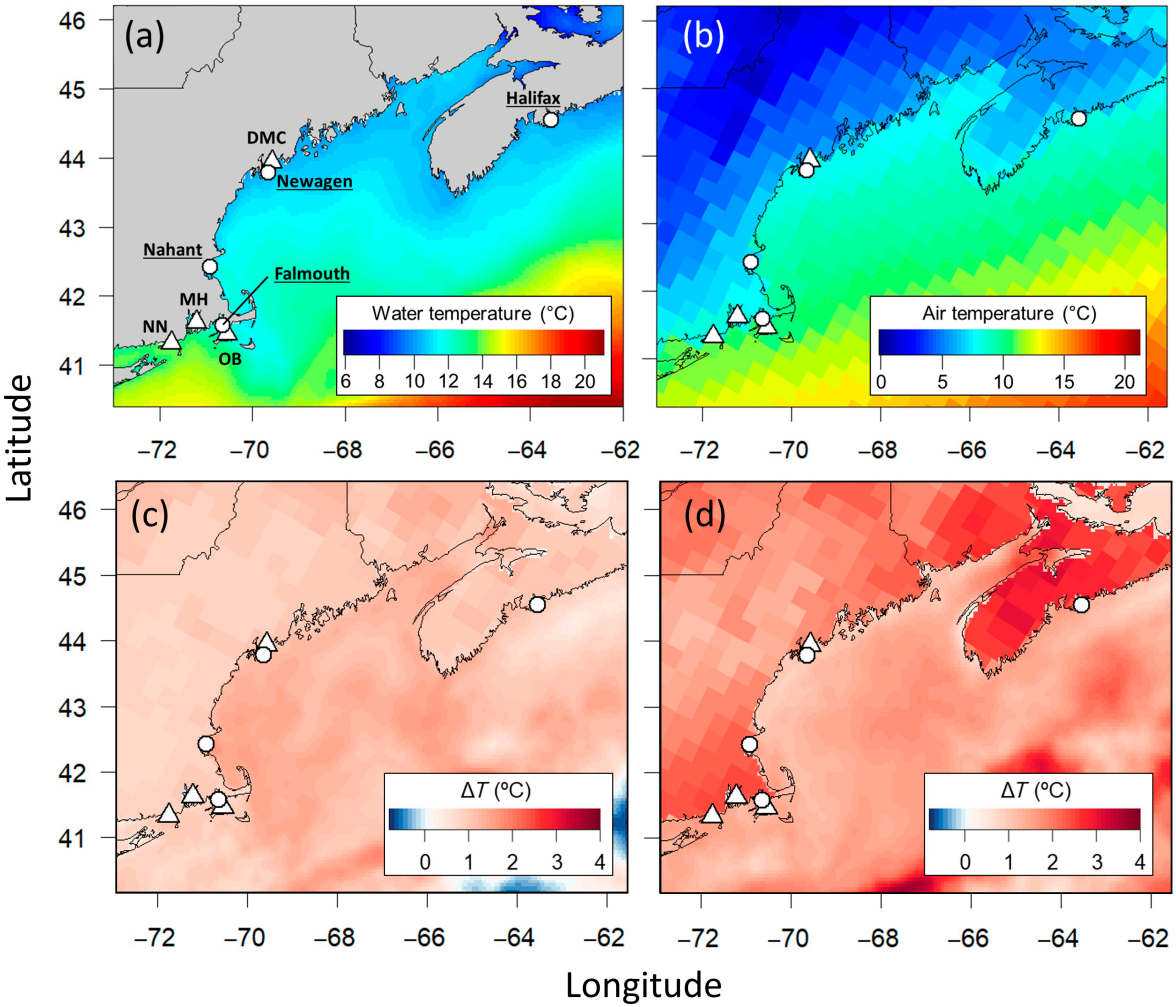
Average sea surface (a) and air (b) temperatures during Oct–Nov–Dec 2019–2023, highlighting spatial gradients. Note that the color scales differ between each panel. Panels (c) and (d) show temperature difference between historical (2001–2005) and modern (2019–2023) sampling periods during Oct–Nov–Dec (c) and Jan–Feb–Mar (d) in both air and water temperatures (with air temperatures displayed over land only). Points denote locations of intertidal field sites. Circles denote sites with barnacle reproduction data collected in both 2002–2004 and 2020–2023, and triangles denote sites where data were collected in 2002–2004 only (Appendix S1: Table S1). Additional data were also collected in Falmouth in 2019–2020 and 2023–2024. Site codes: Damariscotta/the Darling Marine Center (DMC), Oak Bluffs (OB), Mt. Hope (MH), Noyes Neck (NN). Sea surface temperature data source: OSTIA satellite data products (Good et al., 2020). Air temperature data source: NARR climate data products (Mesinger et al., 2006).

We studied intertidal barnacle populations along the northwest Atlantic coast, which exhibits a sharp latitudinal temperature gradient (Figure 1a). Sea surface temperatures (SSTs) and bottom water temperatures both tend to increase from the northeast to the southwest and from the coast to the shelf (Richaud et al., 2016). Air temperatures in the region exhibit less of a latitudinal gradient, with milder temperatures along the coast and cooler temperatures inland (Figure 1b). In contrast to the sharp temperature gradient, differences in fall photoperiod are minimal, peaking at ~20 min difference between our northernmost and southernmost study sites.

Importantly, the northwestern Atlantic is warming faster than almost any other part of the global ocean (Forsyth et al., 2015; Thomas et al., 2017). Air temperatures in the region have also undergone “exceptional warming,” partially induced by the warming trends in SST (Karmalkar & Horton, 2021). We conducted our historical field surveys during relatively cold years while our modern surveys coincided with relatively warm years, allowing us to examine ecological patterns driven by the interannual variation around these long‐term warming trends (Forsyth et al., 2015; Thomas et al., 2017).

### Sample and data collection

*Semibalanus balanoides* reproduction was measured in rocky intertidal habitat at eight sites (Figure 1a) during two historical years (2002–2003 and 2003–2004). Modern sampling was carried out at a subset of four sites (Figure 1a) during three years (2020–2021, 2021–2022 and 2022–2023), with additional sampling at one site during two more modern years (Falmouth, Massachusetts; 2019–2020 and 2023–2024). In two instances, due to limited site access, samples were not collected from the same shore as in the original survey. In these cases, new study sites were established in the same water body, within ~10 km of the original. Locations and study years are listed in Appendix S1: Table S1.

Up to 100 adult *S. balanoides* were collected at each site every 2–4 weeks during the early fall (Sep–Nov), then every 1–2 weeks during the late fall/winter/spring until population‐wide larval release was observed. Individuals were collected haphazardly from across each population's vertical range and horizontal extent to account for heterogeneity within the population. Lone individuals (>1 body length away from other adult barnacles) were excluded from sampling because these are unlikely to be reproductively active (Herrera et al., 2019).

Individuals were evaluated using a dissecting microscope, and their reproductive stage was scored from 0 to 5, based on a simplification of acorn barnacle embryonic development stages described in Crisp (1954) and Anderson (1994) (Table 1). For all analyses, the numbers of adults per sample with each score were converted to proportions to normalize for sample size during weeks when 100 adults could not be collected (due to poor weather or tide conditions). For visualization, embryo scores were aggregated to three categories: non‐brooding adults, adults with early‐stage embryos, and adults with late‐stage embryos (Table 1). For final analyses, embryo scores were further aggregated into adults with and without fertilized embryos (Table 1).

**TABLE 1:** Description of scores used to rate embryonic development of *Semibalanus balanoides*, modified from Crisp (1954) and Anderson (1994).

Fertilization category	Stage	Score	Description
Nonreproductive (unfertilized or post‐brooding)	Non‐brooding	0	No eggs or tissue, or white tissue; white tissue may be “pock‐marked” if larvae were recently released
		1	Yellow tissue that is becoming more egg‐shaped
Fertilized and brooding	Early embryos	2	Eggs visible, ovoid, translucent, and yellow
		3	Eggs with just visible red eye spots
		4	Eggs contain clear, brown eye spot, and nauplii body structure is visible (eye spot and sometimes gut); eggs beginning to turn white
	Late embryos	4b	Nauplii are fully developed (eye spot, gut, and appendages are visible), still contained in eggs
		5	Nauplii are loose under membrane, or swim away when seawater is added

### Phenology analysis

To reveal reproductive phenology patterns, we derived and standardized our reproduction timeseries, estimated the midpoints of fertilization and larval release, and calculated brooding duration for each site and year.

**FIGURE 2:**
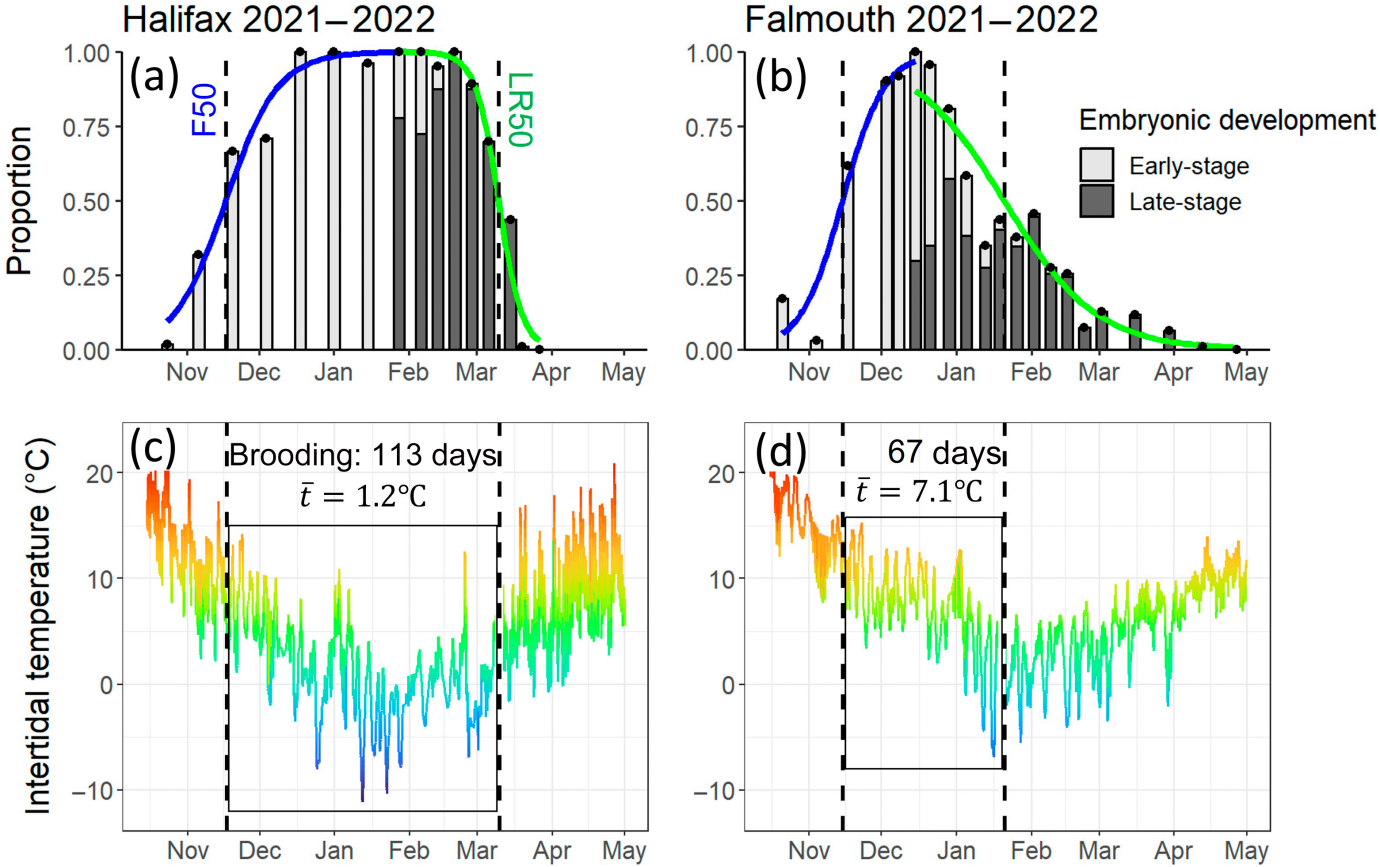
Reproduction phenology of *Semibalanus balanoides* populations in Halifax, Nova Scotia, Canada (a) and Falmouth, Massachusetts, USA (b) in 2021–2022, alongside local intertidal temperatures (c, d). Bars in (a) and (b) indicate the population proportions that contained early‐stage embryos (light gray) and late‐stage embryos (dark gray; Table 1), scaled so that values for fertilized embryos (both early‐ and late‐stage combined; black points) range from 0 to 1. Solid lines denote logistic regression curves, used to characterize fertilization (blue) and larval release (green). Solid lines in (c) and (d) denote modeled intertidal temperatures at each site (see *Methods* 2.5). Dashed lines denote estimated dates of 50% fertilization (F50) and larval release (LR50) at each site. Boxes in (c) and (d) delineate the estimated time for population brooding (F50 to LR50).

We used logistic regression to characterize each transition between brooding and non‐brooding adults, first scaling proportions so that values for brooding adults at each site/year reached 100% (field measurements all included measurements of 0% brooding, so no scaling was needed to standardize minimum values). This ensured that the logistic regression curves were comparable across sites and years and that our analyses of reproduction timing were limited to only those individuals that underwent reproduction (as, in five cases, 10%–21% of adults lacked embryos even during peak brooding, possibly indicating that they were never fertilized). Each series was separated into a fertilization and larval release series, following the increase and decrease, respectively, of brooding adults. For the point of transition, we selected the midpoint of dates when scaled proportions of brooding adults exceeded 95%. This threshold was chosen so that the transition between each fertilization and larval release series corresponded with the approximate midpoint of peak brooding and fitted logistic curves aligned well with field data (assessed visually). Logistic regressions were conducted using the glm function in R (R Core Team, 2021) with a quasi‐binomial error distribution and logit link function. Resulting regression coefficients were used to estimate the date when scaled proportions of brooding adults were equal to 50%, representing the midpoints of fertilization [F50] and larval release [LR50]. Across all years and sites, fertilization and larval release were highly synchronous within each population, and midpoint estimates generally matched scaled field measurements. Time that the population spent brooding embryos was estimated as the number of days between F50 and LR50 (see Figure 2a,b for an example). When field measurements of reproduction began after population‐wide fertilization was already underway, we only used estimates of F50 if the earliest scaled values were <60% fertilized.

In three cases (Halifax 2003–2004 larval release, DMC 2003–2004 fertilization, Nahant 2020–2021 fertilization and larval release), we recorded anomalously low fertilization in the middle of the reproductive season, before late‐stage embryos were observed and, therefore, before larval release could feasibly have occurred (Appendix S1: Figure S4). For these cases, logistic regressions were computed with a smaller subset of the fertilization or larval release timeseries so that regression curves were not skewed by the low fertilization values (see Appendix S1: Section S3).

### Modeled intertidal temperatures

To explore the relationships between intertidal temperature and reproductive phenology, we used the Noah land surface temperature model (Noah LSM v1.91; Ek et al., 2003), modified to simulate intertidal temperature (Wethey et al., 2011) and parameterized for *S. balanoides* body temperature following Herrera et al. (2019). Temperature logger measurements were available for a subset of sites and years, so these were used to assess model accuracy.

Briefly, the modified Noah Intertidal Temperature Model simulates heat transfer between the atmosphere, a biotic layer of *S. balanoides*, and a substrate of solid granite, with periodic tidal flooding. For atmospheric forcing, we used the National Centers for Environmental Prediction (NCEP) North American Regional Reanalysis (NARR) data products (downloaded from https://psl.noaa.gov/) (Mesinger et al., 2006), as in Mislan and Wethey (2011). This included air temperature (°K) at 2 m elevation, downward longwave radiation (W m^−2^), downward shortwave solar radiation (W m^−2^), precipitation rate (kg m^−2^ s^−1^), atmospheric pressure (Pa), relative humidity (%) at 2 m, and wind velocities (m s^−1^) at 10 m, all at a 3‐h timestep and 32‐km spatial resolution (see Figure 1b for example). For ocean temperature forcing, we used the Operational Sea Surface Temperature and Sea Ice Analysis (OSTIA) reprocessed (1981‐09‐30 to 2022‐05‐31) and OSTIA near‐real time (2022‐06‐01 to 2023‐12‐31) satellite data products (Good et al., 2020), which provided daily sea surface temperatures (°K) at a 0.05° spatial resolution (roughly 5 km in the study region; see Figure 1a for example). Sea level height was estimated using the XTide tide prediction software (v2.8.2, https://flaterco.com/xtide/) and harmonics files that extend into Canada (https://flaterco.com/xtide/files.html, 2011‐04‐10, Flater, 2005). Substrate roughness height was set to 5 mm (roughly the height of *Semibalanus* barnacles), and barnacle shell albedo was set to 40% following Herrera et al. (2019). Temperature exchange is simulated between 20 model layers; we use temperatures from “layer 0” (i.e., the topmost layer) to represent shell temperature.

Modeled solar heating and tidal flooding also depend on the orientation of the shore, the slope of the rocky substrate, and the height of the barnacles relative to sea level, which all vary by site. Orientation of the shore at each site was estimated to the nearest 10° using Google Maps. Height of barnacle habitat was estimated as one half of the local mean high water height (i.e., the “mid‐intertidal”). Local substrate slope (either 45, 67.5, or 90°) was selected to match majority of barnacle habitat (summarized in Appendix S1: Table S1). The resulting model output estimated *S. balanoides* body temperature for each site and year at a 30‐min temporal resolution. For all analyses, modeled temperatures were subsampled hourly, and any gaps in the resulting series were linearly interpolated. Hourly values were filtered with the low‐pass PL64 filter (Rosenfeld, 1983) in MATLAB (R2021b) using a cutoff of 33 h, to remove high‐frequency variability (Appendix S1: Figure S2). To assess model precision, we calculated mean error (ME) and root mean square error (RMSE) for the deviation between model predictions and in situ temperature logger measurement data, available for a subset of sites and years (Appendix S1: Figure S3 and Table S2). Logger data were also subsampled hourly and filtered before calculation of error statistics.

### Temperature analysis

To analyze the impacts of temperature on *S. balanoides* phenology, we refer to the following periods: “early fall” (defined as Sep 22 to Nov 20) and “fall” (defined as Sep 22 to Dec 22). To visualize regional atmospheric and ocean warming, we used the gridded NARR air temperature at 2 m and OSTIA SST data products, calculating (1) average temperatures during Oct–Nov–Dec (~fall, simplified for computational purposes) and Jan–Feb–Mar (~winter) during 2001–2005 and 2019–2023, and (2) the temperature change between these two periods.

To explore how fall warming impacted timing of fertilization, we calculated the average fall intertidal temperature at each site across all sampled years and the average date of F50. For each site, we subtracted the year‐specific values from these averages to determine fall temperature anomaly and F50 anomaly, and we compared the values via linear regression. Three fertilization series included subzero temperatures during early fall when fertilization typically occurred (see *Results*). Because cold conditions may also impact the timing of fertilization (Barnes, 1963), these three estimates of F50 were excluded from the regression analysis.

To determine whether intertidal temperatures impacted barnacle brooding, we evaluated brooding duration as a function of average temperature. Intertidal temperatures were subset and averaged for each site and year, according to the estimated start and end dates of brooding (F50 and LR50; see Figure 2c,d for examples). We expected a nonlinear relationship between brooding duration and temperature, so brooding duration estimates were ln‐transformed and then modeled as a function of average (filtered) intertidal temperature during brooding via linear regression. For all analyses, we used *p* < 0.05 as our significance threshold. All analyses were conducted in R (v4.1.1).

## RESULTS

**FIGURE 3:**
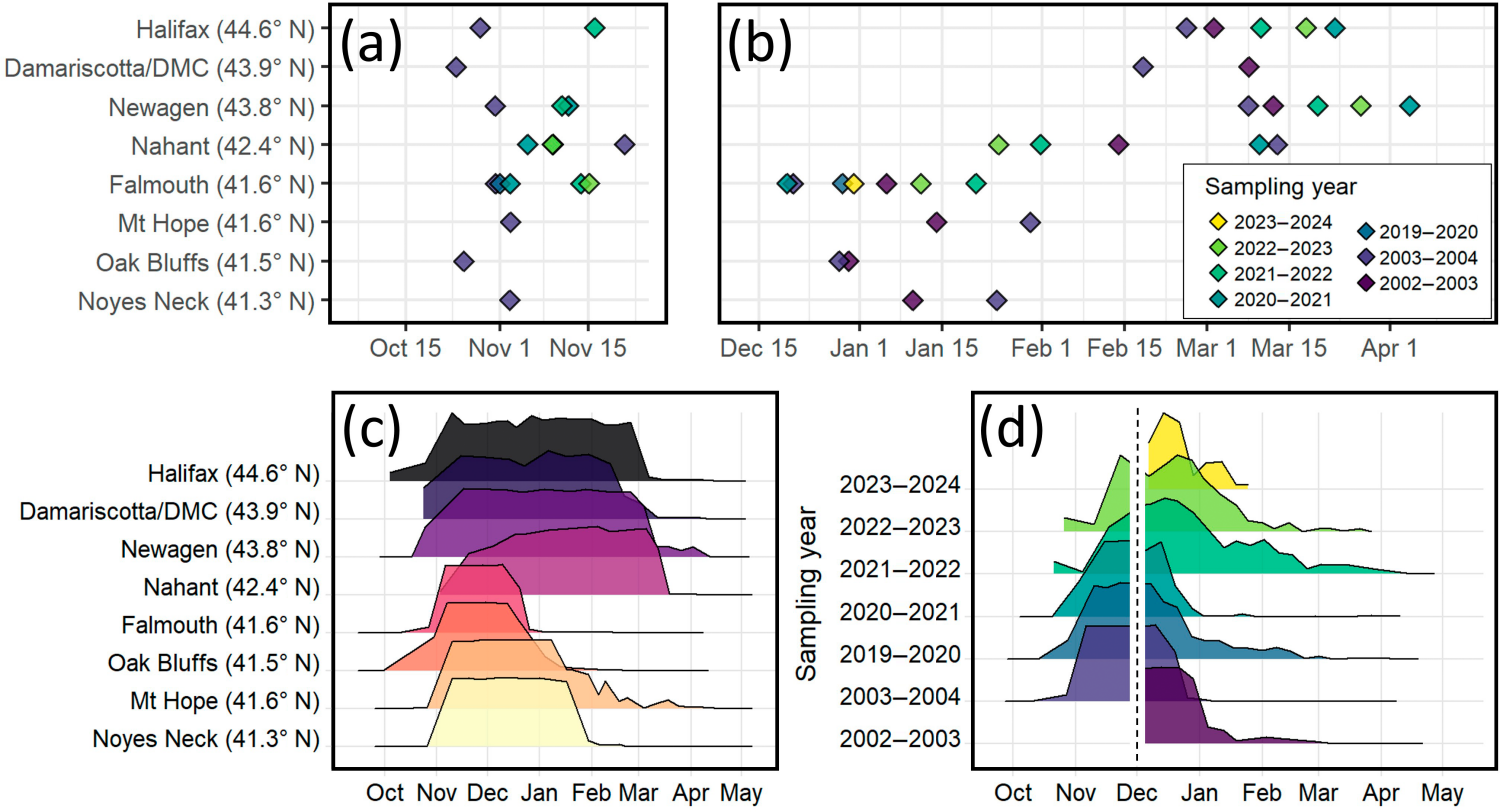
Summary of estimated F50 (a) and LR50 (b) dates for all sampling sites and years. Panels (c) and (d) show a subset of phenology data series, to illustrate the patterns of reproductive timing through space (c) and time (d). Polygons denote the proportion of each population over the course of each year containing fertilized embryos (i.e., early‐ and late‐stages combined; Table 1), scaled so that all series range from 0 to 1. Data in panel (c) are presented for all eight study sites, collected in 2003–2004. Data in panel (d) are presented for Falmouth, Massachusetts, organized chronologically. In the first and last sampling year at Falmouth, sampling began after December 1; a vertical dashed line on Dec 1 was thus added to emphasize that fertilization patterns can only be surmised from the middle 5 years, when sampling began earlier. Colors in panels (a), (b), and (d) denote sampling year. Colors in panel (c) denote sample site.

We found that all *S. balanoides* populations initiated reproduction in the fall. Fertilization (F50) occurred at all sites between Oct 24 and Nov 22, with no consistent latitudinal pattern (Figure 3a). Typically, populations completed fertilization (i.e., proportions increased from ~0% to near‐peak values) in 2–4 weeks, though occasionally this process took up to ~6 weeks (e.g., Nahant in Figure 3c). In 25 out of 30 cases, populations achieved >90% fertilization, and more than half (*n* = 16) achieved 98%–100%. Of the five cases with lower peaks in population‐wide fertilization, two occurred at Nahant (in 2003–2004 and 2022–2023), and three occurred at other sites (Halifax in 2002–2003, Oak Bluffs in 2002–2003, and Falmouth in 2022–2023).

In some cases, fully fertilized populations contained early‐stage embryos for over a month before the appearance of late‐stage embryos (e.g., Figure 2a), while in other cases late‐stage embryos appeared within 2 weeks of peak fertilization (e.g., Figure 2b), indicating that embryonic development rates varied between sites and years. Consequently, some populations in some years (*n* = 10) contained as much as 85%–100% late‐stage embryos, indicating high levels of reproductive activity across the population. Other populations/years (*n* = 7) contained at most 65% late‐stage embryos during any given week of the year, indicating lower reproductive success or possibly a lower degree of fine‐scale synchrony.

**FIGURE 4:**
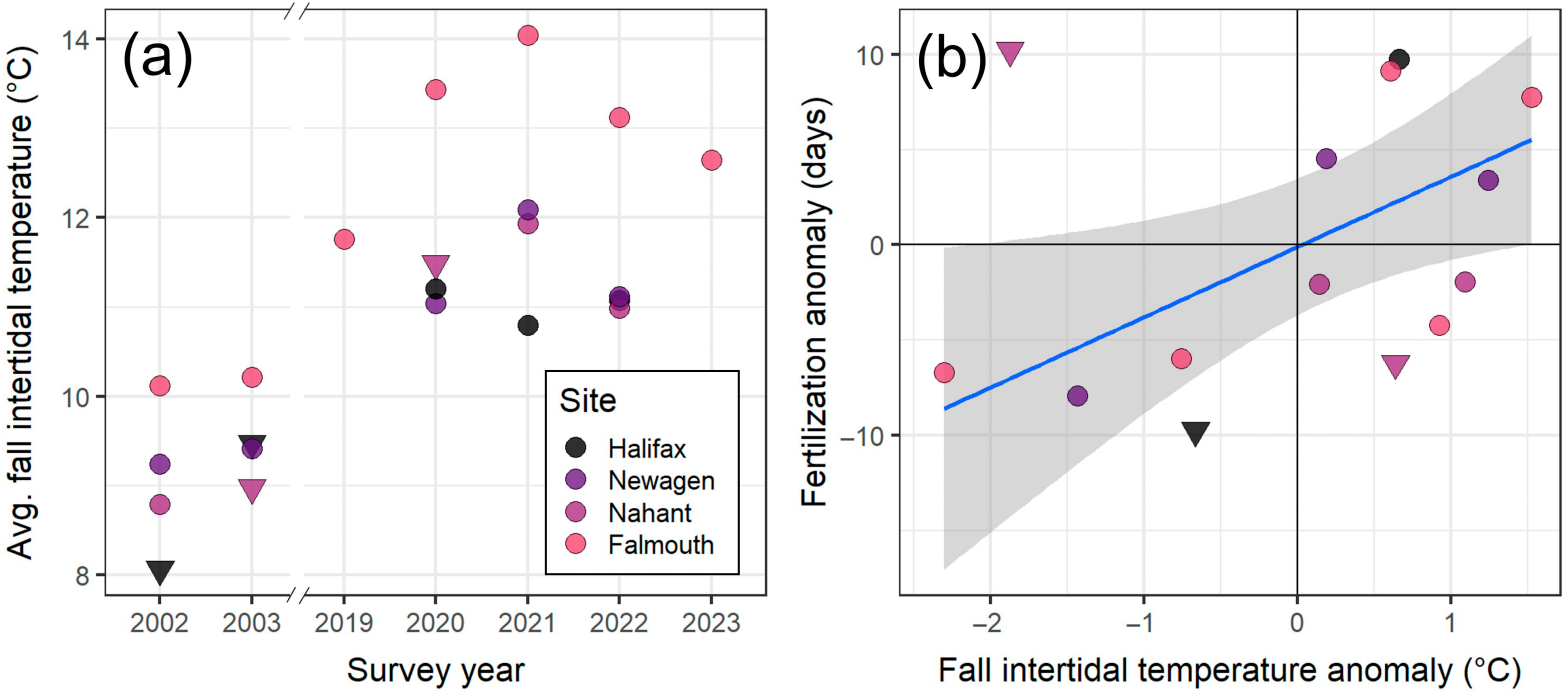
Average fall intertidal temperatures during survey years (a), and the relationship between fall temperature anomaly and fertilization delay (b). Fall temperature anomalies were calculated as the site‐ and year‐specific fall temperature minus the average site‐specific fall temperature across all years with F50 observations. Point shape in (a) and (b) denotes whether filtered temperature series included any subzero values in early fall (triangles). Three such points in panel (b) were excluded from the linear model, because the presence of below‐freezing temperatures may have separately contributed to fertilization timing. The blue line (and gray shading) in panel (b) indicates the best fit linear model (±95% CI).

Intertidal temperatures during modern surveys were substantially warmer than during historical surveys (average 2.4°C warmer in Oct‐Nov‐Dec [OND], with a range of 2.1 to 2.8°C; 3.7°C in Jan‐Feb‐Mar [JFM], with a range of 2.5 to 5.4°C). In OND, when *S. balanoides* fertilization takes place, temperature change between historical and modern years was more pronounced in the Gulf of Maine waters (Figure 1c). In JFM, temperature change was greater overall, particularly in air temperatures around Nova Scotia and southern New England (Figure 1d). Corresponding fall intertidal temperature anomalies significantly correlated with anomalies in F50 (*F*_1,9_ = 7.257; *R* ^2^ = 0.446; *p* = 0.025): At a given site, a warmer fall tended to result in delayed fertilization (3.7 days per 1°C of warming; 95% CI 0.6 to 6.8; Figure 4). Thus, fertilization during modern survey years occurred as much as 20 days later than it had at the same site during historical surveys.

Larval release was highly variable, spanning as little as 1 week to more than 2 months. Across sites, brooding and embryonic development was longer in higher latitude populations than at lower latitude sites (Figures 2 and 3c). Larval release, therefore, varied substantially by latitude: LR50 took place between December 18 and January 29 at sites on Cape Cod and south (Falmouth, Oak Bluffs, Mt. Hope, Noyes Neck), while LR50 occurred between January 24 and April 4 at northern sites (Halifax, Newagen, Damariscotta, Nahant; Figure 3b). This corresponded to brooding durations of 47–88 days at lower latitudes and 76–143 days at higher latitudes.

**FIGURE 5:**
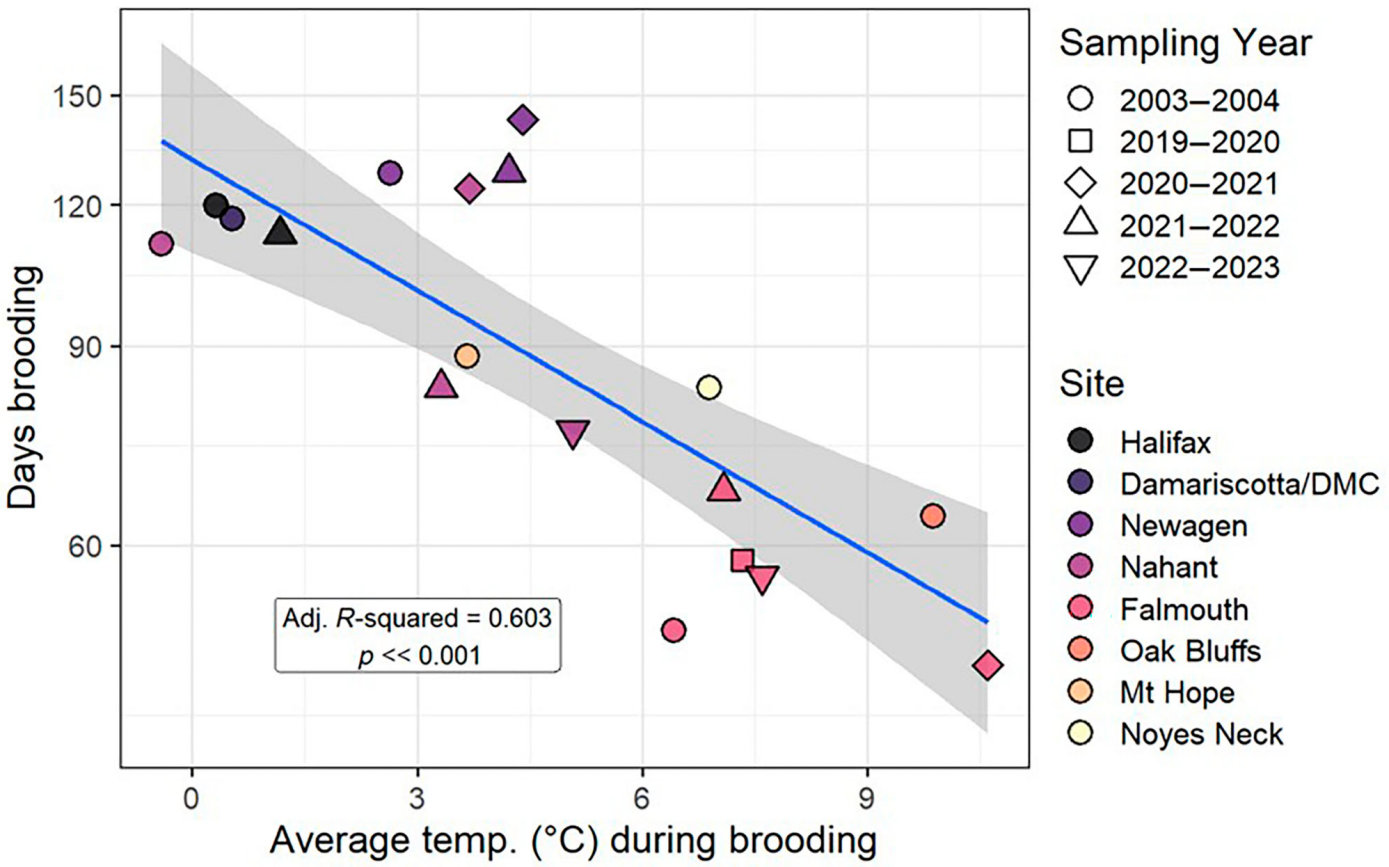
Dependence of brooding duration on intertidal temperatures. Note that the *y*‐axis is log‐scaled. Blue line (and shading) indicates linear model (±95% CI).

Latitudinal differences in brooding duration were largely maintained across years, despite substantial warming throughout the region: the two higher latitude sites (Newagen and Halifax) consistently brooded embryos for >110 days (128–143 days at Newagen, 113–120 days at Halifax), and Falmouth (south of Cape Cod) consistently brooded for <70 days (47–67 days). Only Nahant exhibited highly variable brooding: >110 days in 2 years (112 and 124 days), and <85 days in 2 years (76 and 83 days). Much of the spatial and temporal variation was explained by temperature. Brooding duration decreased exponentially with increasing intertidal temperature (*F*_1,16_ = 26.8; *R* ^2^ = 0.626; *p* << 0.001); warmer temperatures at lower latitudes and, to a lesser extent, in warmer years correlated with much shorter brood duration and, therefore, earlier larval release (Figure 5).

## DISCUSSION

Temperature‐driven patterns and shifts in phenology have been documented in field studies of a wide range of species (Inouye, 2022; Poloczanska et al., 2013), but the consequences of phenology on temperature‐mediated development are seldom explored in natural systems, though these relationships may be key to understanding current and future population connectivity. One such example is the hypothesis that, notwithstanding any effects of phenology, organisms at higher latitudes should experience greater dispersal (Brown, 2014a, 2014b; Janzen, 1967). In terrestrial systems, the seasonal temperature cycle at higher latitudes is thought to facilitate acclimation to a wider range of temperatures, such that dispersal stages will experience higher survivorship despite steep altitudinal temperature clines (Janzen, 1967). In marine systems, the lower average temperatures at higher latitudes should result in slower development rates (Brown, 2014a; O'Connor et al., 2007), giving dispersal stages more time to disperse (Scheltema, 1986; Shanks, 2009). This hypothesis for marine systems was tested with modeled annual mean surface temperature, annual mean current velocity, and frequency of different developmental modes (planktotrophic or lecithotrophic), and potential dispersal was instead predicted to be smallest at midlatitudes (~30° N and S), indicating the importance of spatial variations in physical transport (Álvarez‐Noriega et al., 2020). We show here that phenology may similarly upend simple spatial predictions of dispersal.

**FIGURE 6:**
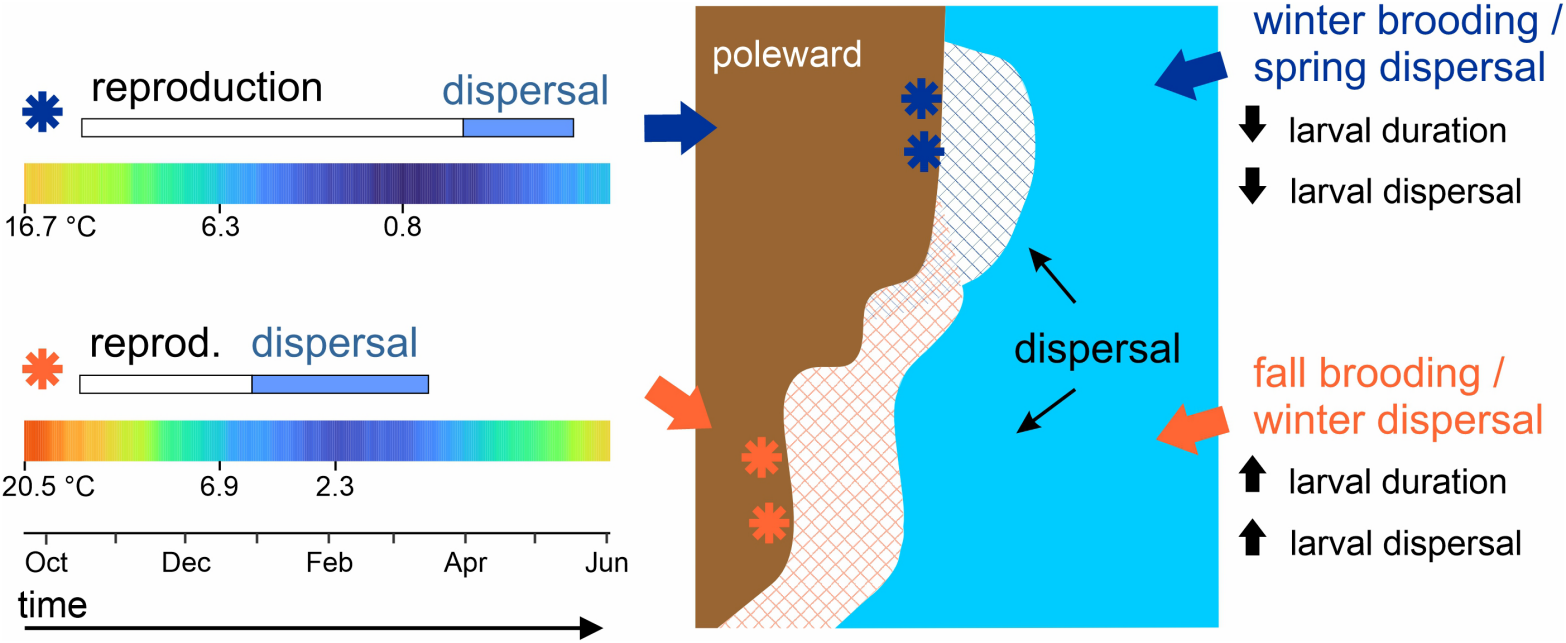
A schematic summary of our findings, along with our predictions for how temperature‐mediated larval release timing may impact dispersal potential in this system. Color bars are 5‐year sea surface temperature climatologies for Halifax and Falmouth, with labels for select dates including maximum and minimum temperatures.

We found that *S. balanoides* brooding duration exponentially decreased with increasing average intertidal temperature, following general expectations for ectotherms (Gillooly et al., 2002), causing lower latitude populations to release larvae up to 3.5 months earlier than higher latitude populations. These differences may have significant impacts on the conditions larvae experience post‐hatching, and they may, counterintuitively, allow for greater dispersal for lower latitude populations. Higher latitude larvae are generally released in spring when waters are warming. These larvae are more likely to experience a more food‐rich environment, and the warmer temperatures will likely speed up larval development (Figure 6). Conversely, lower latitude larvae tend to be released in winter when nearshore waters are still cooling, which may facilitate slower development and greater overall dispersal (Figure 6), provided that biophysical transport processes are comparable across months and sites (Cowen & Sponaugle, 2009).

This regional asynchrony in larval release occurred even though all populations in all years began reproduction within the same 1‐month timeframe. That is, fertilization (F50) displayed little regional or temporal variability despite the wide range of temperature conditions across sites in each year, as was reported in previous field studies (Davenport et al., 2005). However, the timing of fertilization at each site varied year‐to‐year, and we show that this was driven, at least in part, by a combination of warming‐induced delays and disruption by subzero temperatures, although this variation was on the order of ~3 weeks. Latitudinal differences in brooding duration were largely maintained across years, despite substantial warming throughout the region. Only one site (Nahant, Massachusetts) exhibited particularly variable brooding duration (up to 50‐days difference). This site, located between sites to the north and south that exhibited less variation, might represent a transition zone between the two temperature regimes, requiring greater plasticity from barnacle populations to respond to the dominant conditions in any given year.

Many hypotheses have been put forth to explain the possible adaptive advantages of *S. balanoides* reproduction and larval release timing. It has been hypothesized that *S. balanoides* release larvae in response to increased phytoplankton concentrations, so that larvae can access food‐rich waters (Starr et al., 1991). Other studies, however, found that larvae were released in response to turbidity and winter storms, leading the authors to theorize that this behavior facilitates larval transport offshore, to avoid cannibalism and predation on larvae by bottom‐dwelling suspension feeders (Gyory et al., 2013; Gyory & Pineda, 2011). In some of the northernmost populations of *S. balanoides*, larval release must take place before adults are encased in ice for the winter (otherwise resulting in reproductive failure; Peterson, 1966). Ultimately, life cycle event timing is determined by a combination of physiological, behavioral, and evolutionary processes, making the task of predicting future change a challenging one. It follows that even straightforward spatial gradients in temperature can result in complex biological responses and that spatial gradients alone may not suffice for predicting how populations are responding to warming.

Steep temporal and spatial temperature gradients are common in terrestrial and aquatic environments, so any species with broad distributions may exhibit variable reproductive phenology due to temperature‐driven effects on physiology like those described here. Yet, while laboratory studies have shown that processes such as embryonic development are closely tied to temperature (Gillooly et al., 2002), the effects on progeny emergence in natural populations over their distributional range are not well known except in the cases of vital agriculture or fishery species. For example, laboratory studies of the American and Norway lobsters showed that embryonic development shortens with increased temperature, and indeed, warmer coastal temperatures have coincided with earlier onset of hatching in both species (Goldstein & Watson III, 2015; Haarr et al., 2020; McGeady et al., 2021). Similar temperature impacts were found on the growing season of tomatoes in California; the time required for fruit to reach maturity, estimated using a degree‐day model, was longest in the two northern counties, and average growing season decreased over time in all five counties due to warming (Pathak & Stoddard, 2018). Thus, temperature impacts on reproductive physiology and development may have far‐reaching implications for ecosystems, agriculture, and food security, unless organisms (or farmers/fishers) are able to plastically respond to (or predict and plan for) environmental change (e.g., McGeady et al., 2021; Opdal et al., 2024).

Even straightforward spatial gradients in temperature can result in complex biological responses, and spatial gradients alone may not suffice for predicting how populations respond to warming. Despite this, spatial gradients in temperature are often used in studies as a proxy for future warming scenarios in a “space‐for‐time” substitution (Lovell et al., 2023; Pickett, 1989; Raventos et al., 2021; Zografou et al., 2020), but our results corroborate that trends in space and time are not always comparable. We found modest evidence of warming‐induced delays in fertilization, but this did not translate to a latitudinal pattern in fertilization timing. Overall, we emphasize the importance of detailed field measurements collected over long periods of time to disentangle the various processes involved in reproductive phenology and to inform predictions about how ecosystems will change with future warming.

There has been increasing interest in predicting how climate change may impact population dynamics and connectivity (e.g., Fuchs et al., 2020), but predictions are seldom extended to lengthier physiological processes like brooding or fruiting (though see McGeady et al., 2021; Pathak & Stoddard, 2018), and testing with field measurements is needed. We saw evidence of a warming‐induced delay in fall reproduction, consistent with data for other species (Poloczanska et al., 2013 supplementary material). The effect was subtle: <5‐day delay for every degree of warming (Figure 4). Ultimately, phenology shifts due to climate change impacts on physiology will be difficult to predict. Reproduction encompasses several physiological processes that each interact with temperature, during which temperature itself fluctuates (e.g., hourly, daily, fortnightly, seasonally). Moreover, mobile species might also migrate to habitats with different temperature regimes during reproduction and brooding (Cowan et al., 2007; Opdal et al., 2024), adding further complexity to anticipated thermal experience. Lastly, certain aspects of reproduction (e.g., larval release) are behavioral, and changes to the process can be instantaneous (e.g., halting fertilization during particularly cold conditions, releasing larvae in response to turbidity from storms or phytoplankton blooms).

## CONCLUSIONS

As scientists work to predict and plan for ecosystem response to climate change, our findings highlight the difficulty of simple predictions, particularly for regions with substantial spatial and seasonal temperature variation and for organisms whose reproductive cycles span a large portion of that variation. Especially in highly seasonal systems, reproductive phenology controls the food and temperature conditions experienced by progeny, impacting mortality, dispersal, and recruitment (Pineda et al., 2007). Differences in dispersal may in turn impact population recovery from disturbance (Mullineaux et al., 2018), meta‐population stability (Bani et al., 2021), local and regional species diversity (Bowen et al., 2016; Zou & Rudolf, 2023), and the rate of range shifts and expansions of both native and invasive species (Fuchs et al., 2020; Giménez et al., 2020; Poloczanska et al., 2013). We found that expected effects of latitudinal temperature on *S. balanoides* brooding duration may result in counter‐intuitive patterns of dispersal versus latitude, indicating the importance of accounting for the full thermal history of an organism during reproduction in order to accurately predict phenology and the slew of potential knock‐on effects for progeny.

## AUTHOR CONTRIBUTIONS

Jesús Pineda secured funding for the historical sampling, designed the original study, and provided feedback on the analyses, manuscript, and manuscript revisions. Jane B. Weinstock and Jesús Pineda designed the modern sampling. Jane B. Weinstock undertook data processing and analysis, created the tables and figures, and wrote/revised the manuscript. Jesús Pineda and Victoria Starczak developed the simplified embryonic development scores described in Table 1, and Jesús Pineda created Figure 6 with input from Jane B. Weinstock. All authors collected/processed samples and provided input on the manuscript. The present work is part of the doctoral thesis of Jane B. Weinstock.

## CONFLICT OF INTEREST STATEMENT

The authors declare no conflicts of interest.

## Supporting information


Appendix S1.

## Data Availability

Barnacle reproduction data for all sites and years, temperature logger measurements for a subset of sites and years, filtered and unfiltered intertidal temperatures for a subset of sites and years, and example files for use with intertidal temperature model software (Weinstock et al., 2026) are available in Dryad at https://doi.org/10.5061/dryad.gxd2547wk. Code used for data processing, analysis, and visualization (Weinstock, 2024) is available in Zenodo at https://doi.org/10.5281/zenodo.14058404. North American Regional Reanalysis (NARR) climate data are available for download from the National Oceanic and Atmospheric Administration (NOAA) Physical Sciences Laboratory at https://psl.noaa.gov/data/gridded/data.narr.html; this study used 8x daily data for 2001–2005 and 2019–2023 for all variables listed in the *Modeled Intertidal Temperatures* section. Satellite sea surface temperature data are available from Copernicus Marine Service's Marine Data Store at https://data.marine.copernicus.eu/product/SST_GLO_SST_L4_REP_OBSERVATIONS_010_011/; this study used data for 2001–2005 and 2019–2023 for 59°–74° W longitude and 40°–47° N latitude. Noah Land Surface Temperature model software is available from the National Center for Atmospheric Research (NCAR) Research Applications Laboratory at https://ral.ucar.edu/model/unified-noah-lsm. Source code and harmonics files for XTide software are available at https://flaterco.com/xtide/files.html.

## References

[ecy70415-bib-0001] Álvarez‐Noriega, M. , S. C. Burgess , J. E. Byers , J. M. Pringle , J. P. Wares , and D. J. Marshall . 2020. “Global Biogeography of Marine Dispersal Potential.” Nature Ecology & Evolution 4(9): 1196–1203. 32632257 10.1038/s41559-020-1238-y

[ecy70415-bib-0002] Anderson, D. T. 1994. Barnacles: Structure, Function, Development and Evolution. New York, NY: Chapman & Hall.

[ecy70415-bib-0003] Andrew, C. , E. Heegaard , K. Høiland , B. Senn‐Irlet , T. W. Kuyper , I. Krisai‐Greilhuber , P. M. Kirk , et al. 2018. “Explaining European Fungal Fruiting Phenology with Climate Variability.” Ecology 99(6): 1306–1315. 29655179 10.1002/ecy.2237

[ecy70415-bib-0004] Bani, R. , J. Marleau , M. Fortin , R. M. Daigle , and F. Guichard . 2021. “Dynamic Larval Dispersal Can Mediate the Response of Marine Metapopulations to Multiple Climate Change Impacts.” Oikos 130(6): 989–1000.

[ecy70415-bib-0005] Barnes, H. 1963. “Light, Temperature, and the Breeding of *Balanus balanoides* .” Journal of the Marine Biological Association of the United Kingdom 43: 717–727.

[ecy70415-bib-0006] Barnes, H. , and M. Barnes . 1958. “The Rate of Development of *Balanus balanoides* (L.) Larvae.” Limnology and Oceanography 3(1): 29–32.

[ecy70415-bib-0007] Barnes, H. , and M. Barnes . 1976. “The Rate of Development of the Embryos of *Balanus balanoides* (L.) from a Number of European and American Populations and the Designation of Local Races.” Journal of Experimental Marine Biology and Ecology 24: 251–269.

[ecy70415-bib-0008] Barnes, M. 1989. “Egg Production in Cirripedes.” Oceanography and Marine Biology: An Annual Review 27: 91–166.

[ecy70415-bib-0009] Bowen, B. W. , M. R. Gaither , J. D. DiBattista , M. Iacchei , K. R. Andrews , W. S. Grant , R. J. Toonen , and J. C. Briggs . 2016. “Comparative Phylogeography of the Ocean Planet.” Proceedings of the National Academy of Sciences 113(29): 7962–7969. 10.1073/pnas.1602404113 PMC4961182 27432963

[ecy70415-bib-0010] Brown, J. H. 2014a. “Why Are there So Many Species in the Tropics?” Journal of Biogeography 41(1): 8–22. 25684838 10.1111/jbi.12228 PMC4320694

[ecy70415-bib-0011] Brown, J. H. 2014b. “Why Marine Islands Are Farther Apart in the Tropics.” The American Naturalist 183(6): 842–846. 10.1086/676015 24823826

[ecy70415-bib-0012] Conover, D. O. 1992. “Seasonality and the Scheduling of Life History at Different Latitudes.” Journal of Fish Biology 41: 161–178.

[ecy70415-bib-0013] Cowan, D. F. , W. H. Watson , A. R. Solow , and A. M. Mountcastle . 2007. “Thermal Histories of Brooding Lobsters, *Homarus Americanus*, in the Gulf of Maine.” Marine Biology 150(3): 463–470.

[ecy70415-bib-0014] Cowen, R. K. , and S. Sponaugle . 2009. “Larval Dispersal and Marine Population Connectivity.” Annual Review of Marine Science 1(1): 443–466. 10.1146/annurev.marine.010908.163757 21141044

[ecy70415-bib-0015] Crisp, D. J. 1954. “The Breeding of *Balanus Porcatus* (Da Costa) in the Irish Sea.” Journal of the Marine Biological Association of the United Kingdom 33(2): 473–496.

[ecy70415-bib-0016] Crisp, D. J. 1959. “Factors Influencing the Time of Breeding of *Balanus balanoides* .” Oikos 10(2): 275.

[ecy70415-bib-0017] Davenport, J. , M. S. Berggren , T. Brattegard , N. Brattenborg , M. Burrows , S. Jenkins , D. McGrath , et al. 2005. “Doses of Darkness Control Latitudinal Differences in Breeding Date in the Barnacle *Semibalanus balanoides* .” Journal of the Marine Biological Association of the United Kingdom 85(1): 59–63.

[ecy70415-bib-0018] Ek, M. B. , K. E. Mitchell , Y. Lin , E. Rogers , P. Grunman , V. Koren , G. Gayno , and J. D. Tarpley . 2003. “Implementation of Noah Land Surface Model Advances in the National Centers for Environmental Prediction Operational Mesoscale Eta Model.” Journal of Geophysical Research: Atmospheres 108(D22): 8851. 10.1029/2002JD003296.

[ecy70415-bib-0019] Fernández‐Pascual, E. , A. Carta , A. Mondoni , L. A. Cavieres , S. Rosbakh , S. Venn , A. Satyanti , et al. 2021. “The Seed Germination Spectrum of Alpine Plants: A Global Meta‐Analysis.” New Phytologist 229(6): 3573–3586. 33205452 10.1111/nph.17086

[ecy70415-bib-0020] Fischer, G. , F. Ramírez , and F. Casierra‐Posada . 2016. “Ecophysiological Aspects of Fruit Crops in the Era of Climate Change. A Review.” Agronomía Colombiana 34(2): 190–199.

[ecy70415-bib-0021] Flater, D. 2005. “XTide version 2.8.2.” https://flaterco.com/xtide/.

[ecy70415-bib-0022] Forsyth, J. S. T. , M. Andres , and G. G. Gawarkiewicz . 2015. “Recent Accelerated Warming of the Continental Shelf off New Jersey: Observations from the CMVOleander Expendable Bathythermograph Line.” Journal of Geophysical Research: Oceans 120(3): 2370–2384.

[ecy70415-bib-0023] Fuchs, H. L. , R. J. Chant , E. J. Hunter , E. N. Curchitser , G. P. Gerbi , and E. Y. Chen . 2020. “Wrong‐Way Migrations of Benthic Species Driven by Ocean Warming and Larval Transport.” Nature Climate Change 10(11): 1052–1056.

[ecy70415-bib-0024] Gillooly, J. F. , E. L. Charnov , G. B. West , V. M. Savage , and J. H. Brown . 2002. “Effects of Size and Temperature on Developmental Time.” Nature 417(6884): 70–73. 11986667 10.1038/417070a

[ecy70415-bib-0025] Giménez, L. , M. Exton , F. Spitzner , R. Meth , U. Ecker , S. Jungblut , S. Harzsch , R. Saborowski , and G. Torres . 2020. “Exploring Larval Phenology as Predictor for Range Expansion in an Invasive Species.” Ecography 43(10): 1423–1434.

[ecy70415-bib-0026] Goldstein, J. S. , and W. H. Watson, III . 2015. “Influence of Natural Inshore and Offshore Thermal Regimes on Egg Development and Time of Hatch in American Lobsters, *Homarus americanus* .” The Biological Bulletin 228(1): 1–12. 25745096 10.1086/BBLv228n1p1

[ecy70415-bib-0027] Good, S. , E. Fiedler , C. Mao , M. J. Martin , A. Maycock , R. Reid , J. Roberts‐Jones , et al. 2020. “The Current Configuration of the OSTIA System for Operational Production of Foundation Sea Surface Temperature and Ice Concentration Analyses.” Remote Sensing 12(4): 720.

[ecy70415-bib-0028] Gosselin, L. , and P. Qian . 1996. “Early Post‐Settlement Mortality of an Intertidal Barnacle:A Critical Period for Survival.” Marine Ecology Progress Series 135: 69–75.

[ecy70415-bib-0029] Gyory, J. , J. Pineda , and A. Solow . 2013. “Turbidity Triggers Larval Release by the Intertidal Barnacle *Semibalanus balanoides* .” Marine Ecology Progress Series 476: 141–151.

[ecy70415-bib-0030] Gyory, J. , and J. Pineda . 2011. “High‐Frequency Observations of Early‐Stage Larval Abundance: Do Storms Trigger Synchronous Larval Release in *Semibalanus balanoides?* ” Marine Biology 158(7): 1581–1589.

[ecy70415-bib-0031] Haarr, M. L. , M. Comeau , J. Chassé , and R. Rochette . 2020. “Early Spring Egg Hatching by the American Lobster (*Homarus americanus*) Linked to Rising Water Temperature in Autumn.” ICES Journal of Marine Science 77(5): 1685–1697.

[ecy70415-bib-0032] Harms, J. 1984. “Influence of Water Temperature on Larval Development of *Elminius modestus* and *Semibalanus balanoides* (Crustacea, Cirripedia).” Helgoländer Meeresuntersuchungen 38: 123–134.

[ecy70415-bib-0033] Herrera, M. , D. S. Wethey , E. Vázquez , and G. Macho . 2019. “Climate Change Implications for Reproductive Success: Temperature Effect on Penis Development in the Barnacle *Semibalanus balanoides* .” Marine Ecology Progress Series 610(February): 109–123.

[ecy70415-bib-0034] Herrera, M. , D. S. Wethey , E. Vázquez , and G. Macho . 2021. “Living on the Edge: Reproductive Cycle of a Boreal Barnacle at its Southernmost Distribution Limit.” Marine Biology 168(7): 100.

[ecy70415-bib-0035] Inouye, D. W. 2022. “Climate Change and Phenology.” WIREs Climate Change 13(3): e764.

[ecy70415-bib-0036] Janzen, D. H. 1967. “Why Mountain Passes Are Higher in the Tropics.” The American Naturalist 101(919): 233–249.

[ecy70415-bib-0037] Karmalkar, A. V. , and R. M. Horton . 2021. “Drivers of Exceptional Coastal Warming in the Northeastern United States.” Nature Climate Change 11(10): 854–860.

[ecy70415-bib-0038] Lovell, R. S. L. , S. Collins , S. H. Martin , A. L. Pigot , and A. B. Phillimore . 2023. “Space‐for‐Time Substitutions in Climate Change Ecology and Evolution.” Biological Reviews of the Cambridge Philosophical Society 98: 2243–2270. 37558208 10.1111/brv.13004

[ecy70415-bib-0039] McGeady, R. , C. Lordan , and A. M. Power . 2021. “Shift in the Larval Phenology of a Marine Ectotherm Due to Ocean Warming with Consequences for Larval Transport.” Limnology and Oceanography 66(2): 543–557.

[ecy70415-bib-0040] Mesinger, F. , G. DiMego , E. Kalnay , K. Mitchell , P. C. Shafran , W. Ebisuzaki , D. Jović , et al. 2006. “North American Regional Reanalysis.” Bulletin of the American Meteorological Society 87(3): 343–360.

[ecy70415-bib-0041] Mislan, K. A. S. , and D. S. Wethey . 2011. “Gridded Meteorological Data as a Resource for Mechanistic Macroecology in Coastal Environments.” Ecological Applications 21(7): 2678–2690. 22073652 10.1890/10-2049.1

[ecy70415-bib-0042] Mullineaux, L. S. , A. Metaxas , S. E. Beaulieu , M. Bright , S. Gollner , B. M. Grupe , S. Herrera , et al. 2018. “Exploring the Ecology of Deep‐Sea Hydrothermal Vents in a Metacommunity Framework.” Frontiers in Marine Science 5: 49.

[ecy70415-bib-0043] O'Connor, M. I. , J. F. Bruno , S. D. Gaines , B. S. Halpern , S. E. Lester , B. P. Kinlan , and J. M. Weiss . 2007. “Temperature Control of Larval Dispersal and the Implications for Marine Ecology, Evolution, and Conservation.” Proceedings of the National Academy of Sciences 104(4): 1266–1271. 10.1073/pnas.0603422104 PMC1764863 17213327

[ecy70415-bib-0044] Olive, P. J. W. 1992. “The Adaptive Significance of Seasonal Reproduction in Marine Invertebrates: The Importance of Distinguishing between Models.” Invertebrate Reproduction & Development 22(1–3): 165–174.

[ecy70415-bib-0045] Opdal, A. F. , P. J. Wright , G. Blom , H. Höffle , C. Lindemann , and O. S. Kjesbu . 2024. “Spawning Fish Maintains Trophic Synchrony across Time and Space beyond Thermal Drivers.” Ecology 105(6): e4304. 38747119 10.1002/ecy.4304

[ecy70415-bib-0046] Palumbi, S. R. , and M. L. Pinsky . 2013. “Marine Dispersal, Ecology, and Conservation.” In Marine Community Ecology, 2nd ed., edited by M. D. Bertness , J. Bruno , B. R. Silliman , and J. J. Stachowicz . Sunderland, MA: Sinauer Associates.

[ecy70415-bib-0047] Parmesan, C. , and G. Yohe . 2003. “A Globally Coherent Fingerprint of Climate Change Impacts across Natural Systems.” Nature 421(6918): 37–42. 12511946 10.1038/nature01286

[ecy70415-bib-0048] Pathak, T. B. , and C. S. Stoddard . 2018. “Climate Change Effects on the Processing Tomato Growing Season in California Using Growing Degree Day Model.” Modeling Earth Systems and Environment 4(2): 765–775.

[ecy70415-bib-0049] Pau, S. , E. M. Wolkovich , B. I. Cook , T. J. Davies , N. J. B. Kraft , K. Bolmgren , J. L. Betancourt , and E. E. Cleland . 2011. “Predicting Phenology by Integrating Ecology, Evolution and Climate Science.” Global Change Biology 17(12): 3633–3643.

[ecy70415-bib-0050] Peñuelas, J. , T. Rutishauser , and I. Filella . 2009. “Phenology Feedbacks on Climate Change.” Science 324(5929): 887–888. 19443770 10.1126/science.1173004

[ecy70415-bib-0051] Peterson, G. H. 1966. “ *Balanus balanoides* (L.) (Cirripedia) Life Cycle and Growth in Greenland.” Meddelelser om Grønland 159(12): 1–154.

[ecy70415-bib-0052] Philippart, C. J. M. , H. M. van Aken , J. J. Beukema , O. G. Bos , G. C. Cadée , and R. Dekker . 2003. “Climate‐Related Changes in Recruitment of the Bivalve *Macoma balthica* .” Limnology and Oceanography 48(6): 2171–2185.

[ecy70415-bib-0053] Pickett, S. T. A. 1989. “Space‐for‐Time Substitution as an Alternative to Long‐Term Studies.” In Long‐Term Studies in Ecology: Approaches and Alternatives. New York, NY: Springer New York.

[ecy70415-bib-0054] Pineda, J. , J. A. Hare , and S. Sponaugle . 2007. “Larval Transport and Dispersal in the Coastal Ocean and Consequences for Population Connectivity.” Oceanography 20(3): 22–39.

[ecy70415-bib-0055] Poloczanska, E. S. , C. J. Brown , W. J. Sydeman , W. Kiessling , D. S. Schoeman , P. J. Moore , K. Brander , et al. 2013. “Global Imprint of Climate Change on Marine Life.” Nature Climate Change 3(10): 919–925.

[ecy70415-bib-0070] R Core Team . 2021. R: A Language and Environment for Statistical Computing. Vienna: R Foundation for Statistical Computing. https://www.R-project.org/.

[ecy70415-bib-0056] Raventos, N. , H. Torrado , R. Arthur , T. Alcoverro , and E. Macpherson . 2021. “Temperature Reduces Fish Dispersal as Larvae Grow Faster to their Settlement Size.” Journal of Animal Ecology 90(6): 1419–1432. 33508875 10.1111/1365-2656.13435

[ecy70415-bib-0057] Richaud, B. , Y. O. Kwon , T. M. Joyce , P. S. Fratantoni , and S. J. Lentz . 2016. “Surface and Bottom Temperature and Salinity Climatology along the Continental Shelf off the Canadian and U.S. East Coasts.” Continental Shelf Research 124: 165–181.

[ecy70415-bib-0058] Rosenfeld, L. K. 1983. CODE‐1: Moored Array and Large Scale Data Report. Technical Report No. 83–23. Woods Hole, MA: Woods Hole Oceanographic Institution.

[ecy70415-bib-0059] Scheltema, R. S. 1986. “On Dispersal and Planktonic Larvae of Benthic Invertebrates: An Eclectic Overview and Summary of Problems.” Bulletin of Marine Science 39: 33.

[ecy70415-bib-0060] Shanks, A. L. 2009. “Pelagic Larval Duration and Dispersal Distance Revisited.” The Biological Bulletin 216(3): 373–385. 19556601 10.1086/BBLv216n3p373

[ecy70415-bib-0061] Starr, M. , J. H. Himmelman , and J. C. Therriault . 1991. “Coupling of Nauplii Release in Barnacles with Phytoplankton Blooms: A Parallel Strategy to that of Spawning in Urchins and Mussels.” Journal of Plankton Research 13(3): 561–571.

[ecy70415-bib-0062] Staudinger, M. D. , K. E. Mills , K. Stamieszkin , N. R. Record , C. A. Hudak , A. Allyn , A. Diamold , et al. 2019. “It's about Time: A Synthesis of Changing Phenology in the Gulf of Maine Ecosystem.” Fisheries Oceanography 28(5): 532–566. 31598058 10.1111/fog.12429 PMC6774335

[ecy70415-bib-0063] Thomas, A. C. , A. J. Pershing , K. D. Friedland , J. A. Nye , K. E. Mills , M. A. Alexander , N. R. Record , R. Wetherbee , and M. E. Henderson . 2017. “Seasonal Trends and Phenology Shifts in Sea Surface Temperature on the North American Northeastern Continental Shelf.” Elementa: Science of the Anthropocene 5: 48.

[ecy70415-bib-0064] Weinstock, J. 2024. “jbweinstock/Sbal_reproduction: Sbal_reproduction (2024_11_08).” Zenodo. 10.5281/zenodo.14058404.

[ecy70415-bib-0065] Weinstock, J. , J. Pineda , C. DiBacco , S. Genovese , V. Starczak , and K. Krumhansl . 2026. “Data for: Latitudinal variation in seasonal cycle mediates population differences in barnacle reproduction phenology [Dataset].” Dryad. 10.5061/dryad.gxd2547wk. PMC13185677 42153571

[ecy70415-bib-0066] Wethey, D. S. , L. D. Brin , B. Helmuth , and K. A. S. Mislan . 2011. “Predicting Intertidal Organism Temperatures with Modified Land Surface Models.” Ecological Modelling 222(19): 3568–3576.

[ecy70415-bib-0067] White, J. , and J. L. Harper . 1970. “Correlated Changes in Plant Size and Number in Plant Populations.” The Journal of Ecology 58(2): 467.

[ecy70415-bib-0068] Zografou, K. , A. Grill , R. J. Wilson , J. M. Halley , G. C. Adamidis , and V. Kati . 2020. “Butterfly Phenology in Mediterranean Mountains Using Space‐for‐Time Substitution.” Ecology and Evolution 10(2): 928–939. 32015855 10.1002/ece3.5951 PMC6988524

[ecy70415-bib-0069] Zou, H. X. , and V. H. W. Rudolf . 2023. “Priority Effects Determine how Dispersal Affects Biodiversity in Seasonal Metacommunities.” The American Naturalist 202(2): 140–151. 10.1086/725039 37531275

