# Supplementary material for: Latitudinal variation in seasonal cycle mediates population differences in barnacle reproduction phenology
Source: Ecology. 2026 May 19;107:e70415. doi: 10.1002/ecy.70415 (PMC13185677; doi:10.1002/ecy.70415)
Supplement: Supplementary file 1 — Appendix S1. [file ECY-107-e70415-s001.pdf]

# Appendix S1

## Latitudinal variation in seasonal cycle mediates population differences in barnacle reproduction phenology

Jane B. Weinstock, Jesús Pineda, Claudio DiBacco, Salvatore Genovese, Victoria Starczak, and Kira Krumhansl

*Ecology*

### Section S1: Study sites

**Table S1.** Summary of study site locations and intertidal temperature model parameters. We initially modeled temperatures at each site 5 times using 5 different values for slope (0, 22.5, 45, 67.5, and 90°), because many of our sites had multiple habitat characteristics (e.g., boulders on a gently sloping cobble beach; Appendix S1: Figure S1). Generally, an increasing slope resulted in lower maximum temperatures in spring and summer. Temperatures during fall and winter (the periods relevant to this study) and minimum daily temperatures were largely unaffected, so we ultimately selected a slope for each site (either 45, 67.5, or 90°) that best matched the majority of barnacle habitat at each site.

| Site | Location (lat, lon) | Survey years | Angle of shoreline (°) | Slope of substrate (°) | Vertical height (m) |
|---|---|---|---|---|---|
| Halifax,<br>Nova Scotia, Canada | 44.577, -63.550 | 2002-04<br>2020-23 | 110 | 45 | 0.85 |
| Darling Marine Center (DMC),<br>Maine, USA | 43.934, -69.580 | 2002-03 | 270 | 67.5 | 1.44 |
| | 43.936, -69.581 | 2003-04 | 270 | 67.5 | 1.44 |
| Newagen,<br>Maine, USA | 43.787, -69.665 | 2002-04<br>2020-23 | 250 | 45 | 1.44 |
| Nahant,<br>Massachusetts, USA | 42.421, -70.916 | 2002-04 | 260 | 45 | 1.50 |
| | 42.417, -70.929 | 2020-23 | 180 | 90 | 1.50 |
| Falmouth,<br>Massachusetts, USA | 41.642, -70.651 | 2002-04 | 270 | 67.5 | 0.65 |
| | 41.532, -70.671 | 2019-24 | 10 | 67.5 | 0.65 |
| Oak Bluffs (OB),<br>Massachusetts, USA | 41.461, -70.557 | 2002-04 | 150 | 67.5 | 0.40 |
| Mount Hope (MH),<br>Rhode Island, USA | 41.626, -71.216 | 2002-04 | 190 | 67.5 | 0.55 |
| Noyes Neck (NN),<br>Rhode Island, USA | 41.327, -71.754 | 2002-04 | 180 | 67.5 | 0.42 |

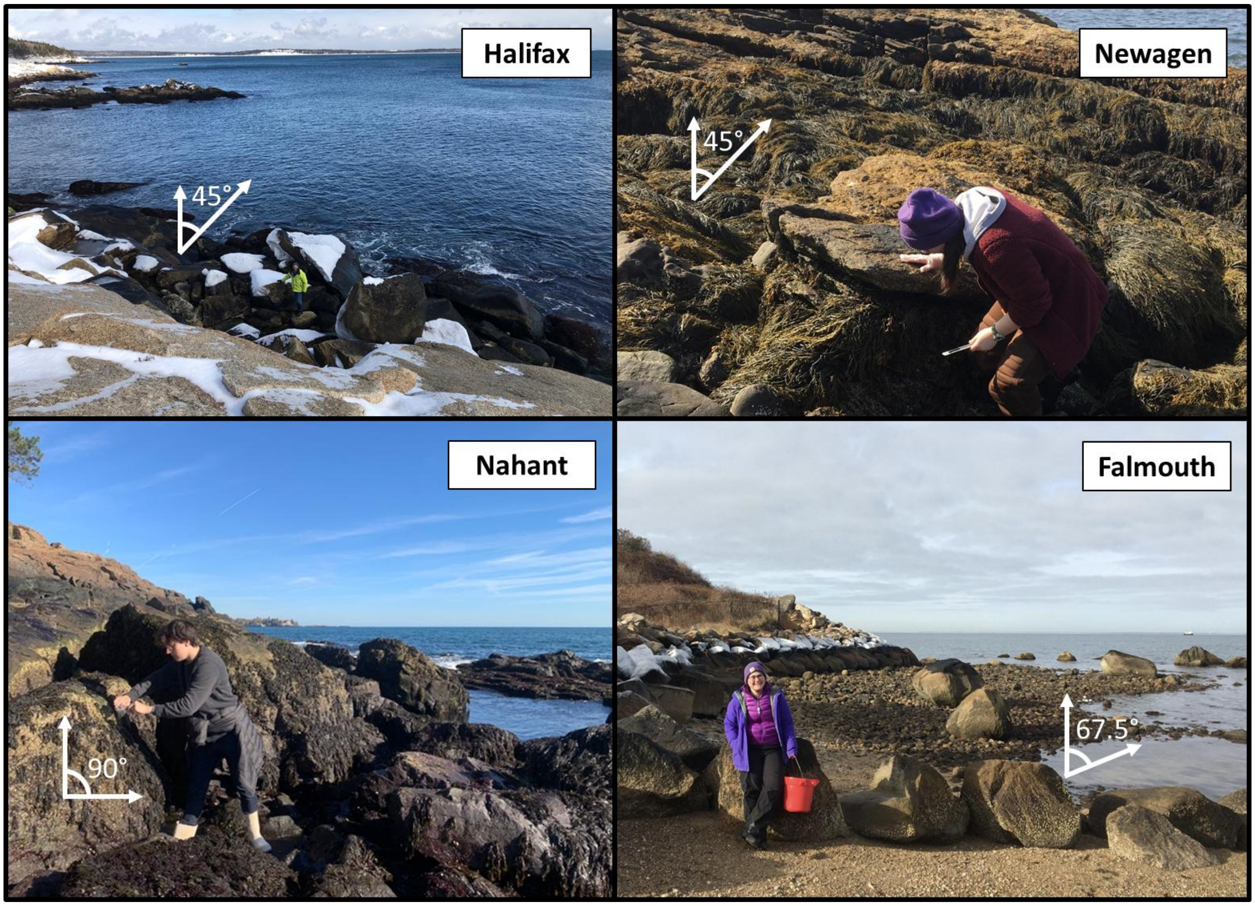


**Figure S1.** Pictures of our four modern field sites, illustrating habitat complexity. Angles used in intertidal temperature modeling were added to each picture, in white. Photo credits: Halifax photo by Catherine Johnson (used with permission); Newagen and Falmouth photos by Aaron Meneghini (used with permission); Nahant photo by Salvatore Genovese.

**Supporting field stations, marine labs, and research institutions:** Samples and sample processing were conducted at:

The Fisheries and Oceans Canada Bedford Institute of Oceanography in Dartmouth, Nova Scotia, Canada

The University of Maine Darling Marine Center in South Bristol, Maine, U.S.A.

The Northeastern University Marine Science Center in Nahant, Massachusetts, U.S.A.

Boston University in Boston, Massachusetts, U.S.A.

The Woods Hole Oceanographic Institute in Woods Hole, Massachusetts, U.S.A.

**Section S2: Intertidal temperature modeling**

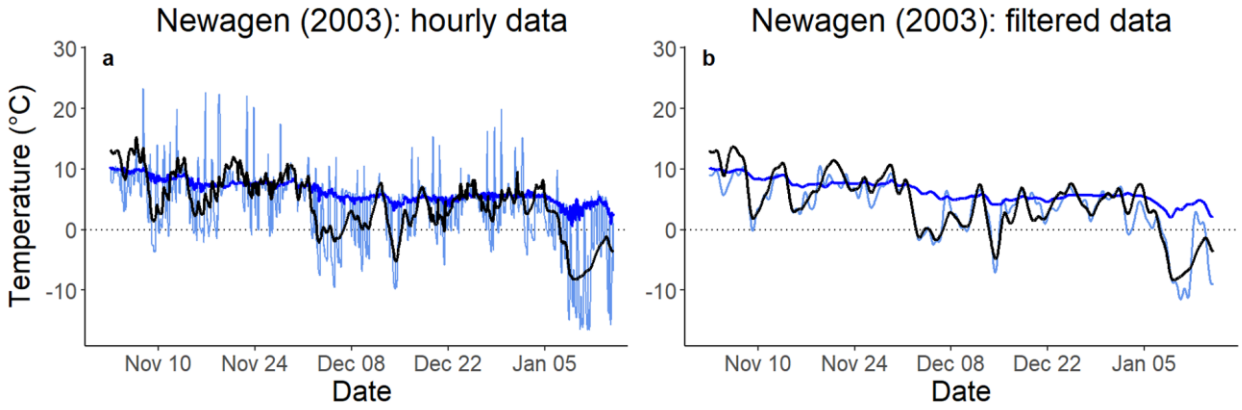


**Figure S2.** Example of the NOAH Intertidal Model output (**black**), as compared to a locally placed intertidal temperature logger (**light blue**) and subtidal temperature logger (**dark blue**). Panel **a** shows hourly temperature series, while panel **b** shows filtered series. Tidbit temperature loggers (Onset corp., Bourne, Massachusetts, USA) were placed in areas of dense barnacle cover at all eight study sites during 2002-04, allowing for a direct assessment of model performance at each site. Because many studies of intertidal ecology use water temperature measured away from the shore, we also included subtidal logger-measured temperatures.

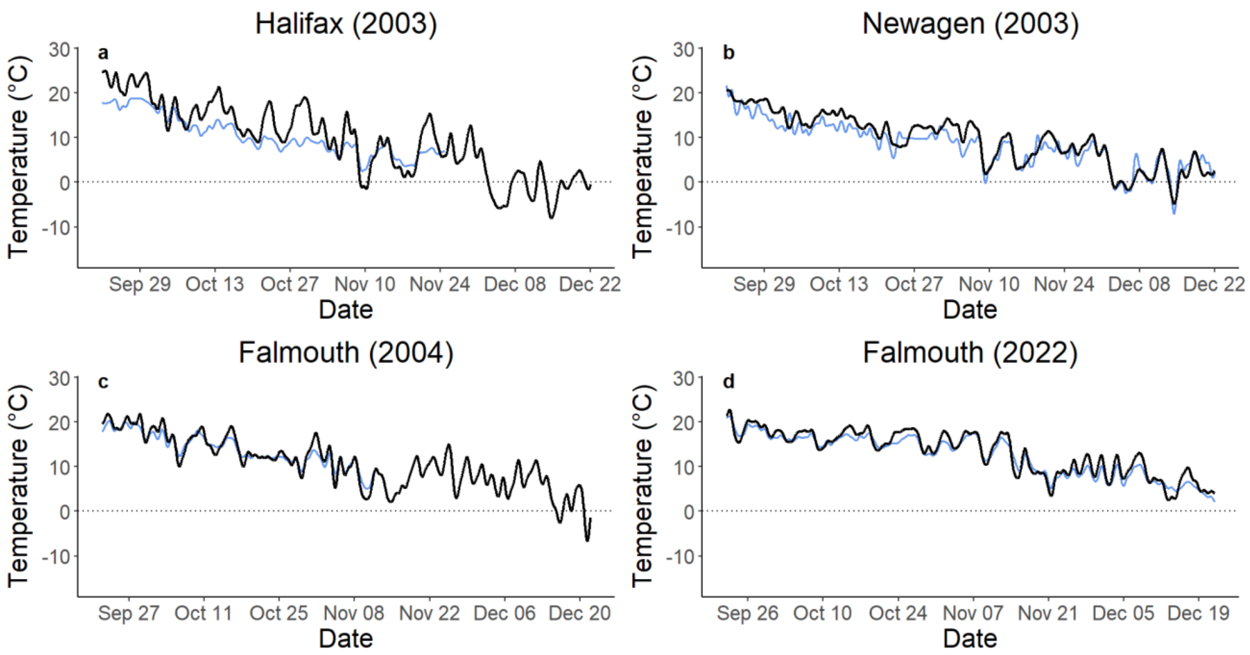


**Figure S3.** Filtered NOAH Intertidal Model output (**black**) and filtered intertidal temperature logger data (**light blue**) for fall months at four site-year combinations. Note that temperature logger data does not extend for the full fall period in two instances (Halifax 2003 [a] and Falmouth 2004 [c]).

**Table S2.** Error statistics of filtered Noah Intertidal Temperature model relative to filtered temperature measurements collected by locally placed Tidbit temperature loggers (Onset corp., Bourne, Massachusetts, USA). ME = mean error, RMSE = root mean square error, *n* = number of hourly observations

| | ME | RMSE | *n* |
|---|---|---|---|
| Halifax 2003 | -1.12 | 4.06 | 1532 |
| Newagen 2003 | -2.68 | 2.13 | 2108 |
| Falmouth 2004 | -0.304 | 1.37 | 1218 |
| Falmouth 2022 | -0.754 | 1.37 | 2208 |

**Section S3: Cases of low fertilization mid-season**

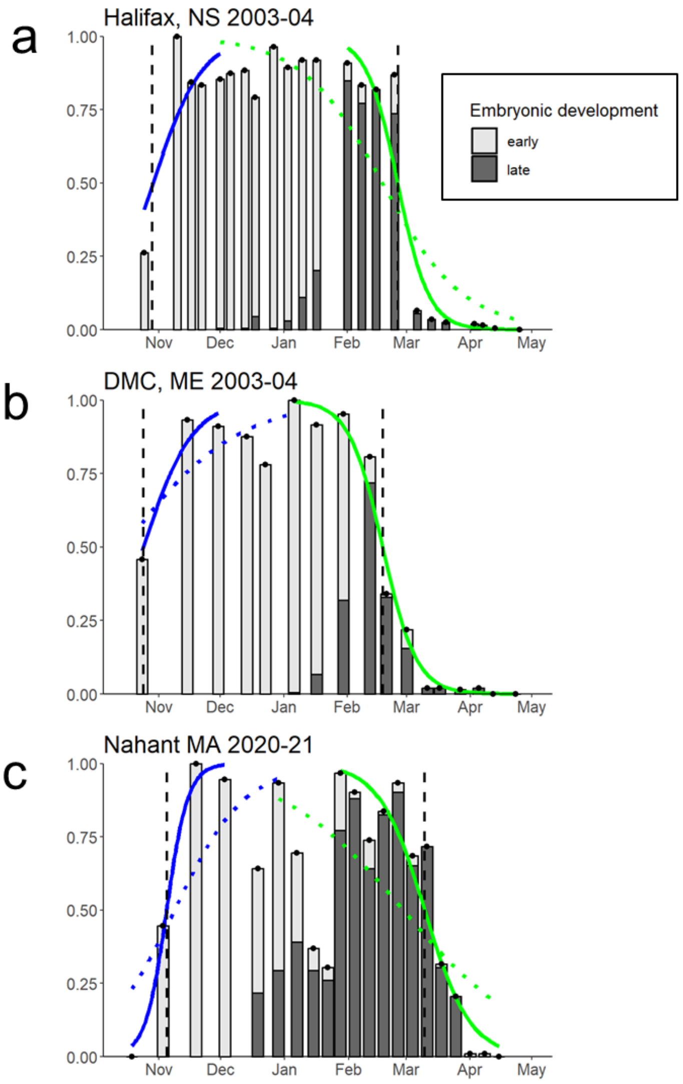


**Figure S4.** Reproduction data for *Semibalanus balanoides* populations in Halifax, Nova Scotia, Canada in 2003-04 (**a**), the Darling Marine Center, Maine, USA in 2003-04 (**b**), and Nahant, Massachusetts, USA in 2020-21 (**c**). Bars indicate the proportion of the population that contained early-stage embryos (light grey) and late-stage embryos (dark grey; Table 1), scaled so that values containing fertilized embryos (both early- and late-stage combined; black points) range from 0 to 1. Dashed lines denote automated logistic regression curves, intended to characterize fertilization (**blue**) and larval release (**green**). Solid lines denote revised logistic curves, carried out on subsets of each data series so that interpolated values of 50% fertilization and larval release (vertical dashed lines) better matched the actual data. In panels **a** and **b**, it is possible that one week of sampling happened to include particularly high numbers of fertilized adults, which served to skew the data scaling and make mid-season values low by comparison. In panel **c**, the low mid-season values may have been an artifact of sampling (e.g., if poor weather/tide conditions limited

sampling to high-intertidal individuals), or they may be indicative of higher levels of patchiness in the reproductive processes of Nahant barnacles during the 2020-21 reproductive season.
